# Function of Plant DExD/H-Box RNA Helicases Associated with Ribosomal RNA Biogenesis

**DOI:** 10.3389/fpls.2018.00125

**Published:** 2018-02-08

**Authors:** Yuelin Liu, Ryozo Imai

**Affiliations:** Institute of Agrobiological Sciences, National Agriculture and Food Research Organization, Tsukuba, Japan

**Keywords:** nucleolus, DExD/H-box RNA helicase, ribosome, development, stress response

## Abstract

Ribosome biogenesis is a highly complex process that requires several cofactors, including DExD/H-box RNA helicases (RHs). RHs are a family of ATPases that rearrange the secondary structures of RNA and thus remodel ribonucleoprotein complexes. DExD/H-box RHs are found in most organisms and play critical roles in a variety of RNA-involved cellular events. In human and yeast cells, many DExD/H box RHs participate in multiple steps of ribosome biogenesis and regulate cellular proliferation and stress responses. In plants, several DExD/H-box RHs have been demonstrated to be associated with plant development and abiotic stress tolerance through their functions in modulating pre-rRNA processing. In this review, we summarize the pleiotropic roles of DExD/H-box RHs in rRNA biogenesis and other biological functions. We also describe the overall function of the DExD/H-box RH family in ribosome biogenesis based on data from human and yeast.

## Introduction

The ribosome is an important molecular machine where mRNA is translated into protein. In eukaryotes, the ribosome consists of a small 40S and a large 60S ribonucleoprotein (RNP) subunit. Ribosome biogenesis is a highly complex and fundamental process that comprises the maturation of rRNAs and their assembly with ribosomal proteins (RPs; Panse and Johnson, 2010). Biosynthesis of rRNA initiates with the transcription of 35S pre-rRNA and 5S rRNA by RNA polymerase I and RNA polymerase III respectively, followed by multiple steps of pre-rRNA processing, modification and folding to yield mature rRNAs (Henras et al., 2008; Woolford and Baserga, 2013). A large number of non-coding RNA and ribosome biogenesis factors (RBFs), such as endo- and exoribonuclease, rRNA-modifying enzymes, and RNA helicases (RHs), participate in these processes (Kressler et al., 2010; Lafontaine, 2015). In human cells, mutations in RBFs and RPs usually cause defects in pre-rRNA processing, resulting in various genetic disorders (Narla and Ebert, 2010; Tafforeau et al., 2013). In plants, RBFs and RPs modulate growth and development, affecting processes including leaf morphology, gametogenesis, and embryo development (Byrne, 2009; Weis et al., 2015).

RHs are enzymes that rearrange the secondary structure of RNA and RNP complexes in an energy-dependent manner (Tanner and Linder, 2001). RHs are classified into six subclasses, SF1–SF6, based on specific motif sequences (Gorbalenya and Koonin, 1993; Singleton et al., 2007). SF2 is the major subclass of RHs, and its members share 12 conserved motifs that are involved in ATP hydrolysis, RNA binding, and intramolecular interactions (Fairman-Williams et al., 2010; Linder and Fuller-Pace, 2013). The related DEAH-, DExD-, and DExD/H-Box families are often referred to as the DExD/H family. They, together with the DEAD-Box family, constitute the main group of SF2 RHs (Gorbalenya and Koonin, 1993; Fairman-Williams et al., 2010). DExD/H-box RHs are involved in most cellular events associated with RNA, such as ribosome biogenesis, spliceosome assembly, RNA decay, and RNA editing (Jarmoskaite and Russell, 2014). Recent research has revealed the importance of DExD/H-box RHs in many aspects of plant RNA metabolism and physiological processes. In this review, we will focus on the emerging roles of plant nucleolus DExD/H-box RHs in rRNA biogenesis, and summarize the current research on their functions in growth and stress responses.

## Yeast and Human DExD/H-Box RHs in Ribosome Biogenesis

Over the past several decades, the functions of DExD/H-box RHs have been widely studied in yeast and human cells. Approximately half of the characterized RHs are associated with various steps in ribosome biogenesis, from rRNA transcription to final ribosome subunit maturations (Martin et al., 2013; Rodríguez-Galán et al., 2013). Most yeast DExD/H-box RHs involved in ribosome biogenesis are essential for cell viability (Rocak and Linder, 2004). However, some are non-essential and their mutants display specific phenotypes. The yeast mutant of *Dbp2*, which functions in both nonsense-mediated mRNA decay and 25S rRNA biogenesis (Bond et al., 2001), displays growth retardation under cold conditions (Barta and Iggo, 1995). Disruptions of *Dbp3* and *Dbp7*, two additional RHs involved in pre-rRNA processing, render a slow growth phenotype under both optimal and cold conditions (Weaver et al., 1997; Daugeron and Linder, 1998). In human cells, deregulation of ribosome-biogenesis-related DExD/H-box RHs is associated with tumorigenesis. DDX21 is highly expressed in breast carcinomas; it can promote tumorigenesis by enhancing RNA processing in breast cancer cells (Zhang et al., 2014). DDX10 is associated with the inv(11)(p15q22) chromosome abnormality which is found in some myeloid leukemia patients (Arai et al., 1997). The NUP98–DDX10 chimeric protein may promote tumorigenesis through altered ribosome assembly or aberrant mRNA transportation (Arai et al., 1997). DDX5 and DDX17 have pro-proliferation or oncogenic functions in cancer development. They are up-regulated in different types of cancer cells, such as colon, prostate, and cutaneous squamous cell carcinoma (Fuller-Pace and Moore, 2011; Fuller-Pace, 2013). Together, these studies suggest that yeast and human DExD/H-box RHs that are involved in ribosome biogenesis play important roles in maintaining cell proliferation as well as stress adaptation.

## Biological Roles of Ribosome Biogenesis Related DExD/H-Box RHs in Plants

Plant genomes encode a larger and more diverse DExD/H RH family than is found in other organisms (Linder and Owttrim, 2009; Xu et al., 2013). Recently, increasing numbers of plant DExD/H-box RHs have been functionally characterized and their roles in biotic and abiotic stresses as well as plant development have been extensively studied (**Table 1**). Among these DExD/H-box RHs, only a small fraction is known to be involved in ribosome biogenesis. AtRH36/SWA3, a homolog of yeast Dbp8p, is the first ribosome biogenesis-related RH that has been functionally analyzed. Knockdown mutants of *AtRH36* display higher accumulations of immature rRNA precursors than WT (Huang et al., 2010a). Loss-of-function of *AtRH36* resulted in a disrupted progression of mitosis during female gametophyte development, whereas the RNAi knock-down mutants displayed several defects in growth and development, such as short roots and abnormal leaves (Huang et al., 2010a; Liu et al., 2010). AtRH18 is another essential DExD/H-Box RH involved in ribosome biogenesis. AtRH18 is a homolog of yeast Sbp4p which participates in the biogenesis of the 60S ribosomal subunit (de la Cruz et al., 1999). Plants with reduced AtRH18 activity show chlorosis, while the knockout mutants are embryo-lethal (Plötner et al., 2017).

**Table 1:** List of characterized DEAD-box RHs in *Arabidopsis*, rice, and maize.

Plant	Symbol	RNA metabolism	Physiological function	Reference
*Arabidopsis*	AtRH3	Chloroplast ribosome biogenesis	Chloroplast development	Asakura et al., 2012
		roup II intron splicing	ABA response	Gu et al., 2014
			Stress tolerance	Lee et al., 2013
	AtRH7	Ribosome biogenesis	Plant development	Huang et al., 2016
			cold tolerance	Liu et al., 2016
	AtRH10	Ribosome biogenesis	High-temperature tolerance	Matsumura et al., 2016
	AtRH22	Chloroplast ribosome biogenesis	Chloroplast development	Chi et al., 2012
			Seed oil biosynthesis	Kanai et al., 2013
	AtRH36	Ribosome biogenesis	Female gametogenesis	Huang et al., 2010a
				Liu et al., 2010
	AtRH39	Chloroplast ribosome biogenesis	Chloroplast development	Nishimura et al., 2010
	LOS4	mRNA export	Stress tolerance	Gong et al., 2002, 2005
	PMH2	Group II intron splicing	Unidentified	Köhler et al. (2010)
	RCF1	mRNA splicing	Freezing tolerance	Guan et al., 2013
	STRS1	Gene silencing	Stress tolerance	Kant et al., 2007
	STRS2			Khan et al., 2014
	ESP3	mRNA splicing	Embryonic development	Herr et al., 2006
	RID1	mRNA splicing	Root development	Ohtani et al., 2013
	AtRH57	Ribosome biogenesis	Glucose and ABA response	Hsu et al.,2014
	ABO6	Mitochondrial RNA splicing	ABA and auxin signaling	He et al., 2012
	HEN2	RNA surveillance	Flower development	Western et al., 2002;
				Lange et al., 2014
	AtMTR4	Ribosome biogenesis	Plant development	Lange et al., 2011
	ISE2	Chloroplast ribosome biogenesis	Plasmodesmata regulation	Carlotto et al., 2016
		Group II intron splicing		Bobik et al., 2017
	AtRH2	Unidentified	Tombusvirus defense	Kovalev and Nagy, 2014
	AtRH8	Unidentified	Potyviruses defense	Huang et al., 2010c
	AtRH9	Unidentified	Potyviruses defense	Li et al., 2016
	AtRH18	Unidentified	Embryonic development	Plötner et al., 2017
	AtRH20	Unidentified	Tombusvirus defense	Kovalev et al., 2012
	ISE1	Unidentified	Plasmodesmata regulation	Stonebloom et al., 2009
	AtHELPS	Unidentified	K^+^ tolerance	Xu et al., 2011
Rice	TOGR1	Ribosome biogenesis	Thermotolerance	Wang et al., 2016
	OsRH36	Unidentified	Female gametogenesis	Huang et al., 2010b
	OsBIRH1	Unidentified	Biotic and abiotic stress tolerance	Li et al., 2008
	OsSUV3	Unidentified	Salt tolerance	Tuteja et al., 2013
Maize	ZmRH3	Chloroplast ribosome biogenesis	Unidentified	Asakura et al., 2012
		Group II intron splicing		
	ZmDRH1	Ribosome biogenesis	Unidentified	Gendra et al., 2004

Knocking out of non-essential RHs resulted in pleiotropic phenotypes. AtMTR4 is a predominantly nucleolar localized DExD/H protein that associates with the RNA exosome complex and functions in rRNA maturation and surveillance (Lange et al., 2011, 2014). A mutation in *AtMTR4* increases accumulation of rRNA precursors and rRNA maturation by-products, resulting in several developmental defects, such as delayed embryogenesis, abnormal cotyledons, shorter root, etc. (Lange et al., 2011, 2014). It has been reported that AtRH57 is an ATP-independent RH, and that it can be induced by glucose, ABA, and salt (Hsu et al., 2014). Functional analyses using *atrh57* knockout mutants indicated that AtRH57 negatively regulates glucose-mediated ABA accumulation and signaling in germination and early seedling development (Hsu et al., 2014). In addition, *AtRH57* mutations cause accumulation of abnormal rRNA precursors, hampering small ribosomal subunit formation, which becomes more significant with high levels of glucose (Hsu et al., 2014). Recently, the relationship between DExD/H-box RHs and temperature stress tolerance has been reported in *Arabidopsis*. AtRH10 is a homolog of human DDX47 and yeast Rrp3p, both of which are involved in ribosome biogenesis (Matsumura et al., 2016). The missense *rh10* mutant, impaired in pre-rRNA processing, shows defects in primary root elongation and leaf development under high temperature (Matsumura et al., 2016; Ohbayashi et al., 2017). AtRH7/PRH75 is a bifunctional RH with RNA unwinding and rewinding activities (Nayak et al., 2013) and is required for pre-rRNA processing (Huang et al., 2016; Liu et al., 2016). The *rh7* mutants display pleiotropic phenotypes in growth and development including pointed leaves and disturbed vascular patterns which are also found in ribosome-related mutants (Huang et al., 2016; Liu et al., 2016). The functions of AtRH7 are associated with low-temperature stress. *rh7* mutants exhibit severe retardation in germination and defects in leaf morphogenesis under a mild cold stress condition (12°C), and cannot survive under prolonged 4°C treatment (Huang et al., 2016; Liu et al., 2016). Genetic analysis showed that the abnormal accumulation of rRNA precursors in *rh7* was elevated when these plants were exposed to cold (Huang et al., 2016; Liu et al., 2016); thus, cold may trigger the abnormal phenotypes in the mutant. Interestingly, AtRH7 can physically interact with AtCSP3 (Cold Shock Domain Protein 3), an RNA chaperone involved in cold adaptation (Kim et al., 2013), suggesting that AtRH7 may complex with AtCSP3 to regulate the secondary structure of pre-rRNA, and thus ensure proper pre-rRNA processing in *Arabidopsis*.

Functions of DExD/H-box RHs as RBFs were also investigated in crop plants. OsRH36 complemented the homologous *atrh36-1* mutant and was required for either gametogenesis or fertilization during reproduction in rice (Huang et al., 2010b). Recently, another rice DExD/H-box RH, TOGR1 (Thermotolerant Growth Required 1), was isolated by map-based cloning from a thermosensitive dwarf *indica* mutant (Wang et al., 2016). The *togr1* mutant exhibited high-temperature-dependent dwarf phenotypes. Overexpression of *TOGR1* resulted in enhanced thermotolerance as well as increased plant height and yield under high-temperature conditions (Wang et al., 2016). Molecular analyses on the *togr1* mutant demonstrated that TOGR1 is associated with U3 snoRNA and is involved in pre-rRNA homeostasis (Wang et al., 2016). When the temperature was raised from 25 to 38°C, rRNA precursor *P-A3* accumulated to a higher level in *togr1* mutants than in WT, suggesting a crucial role of TOGR1 in maintaining pre-rRNA processing at high temperatures (Wang et al., 2016). In maize, the DExD/H-box RH, ZmDRH1, has been shown to interact with MA16 and fibrillarin to form a RNP complex involved in rRNA metabolism (Gendra et al., 2004). Collectively, the recent studies of plant DExD/H-box RHs have revealed multiple roles for these enzymes in plant development and stress adaptation.

## Potential Players in Ribosome Biogenesis

Considering the fact that about half of the identified DExD/H-box RHs from human and yeast are RBFs (Rocak and Linder, 2004; Jankowsky et al., 2011; Rodríguez-Galán et al., 2013), we reasoned that there are more DExD/H-box RHs associated with rRNA metabolism in plants than have previously been identified. We thus conducted a database search using *Ensembl Plants*^1^ to identify the homologs of human and yeast DExD/H-box RHs from *Arabidopsis*, rice and maize genomes. Twenty-eight potential candidates, whose homologs in human and yeast were involved in ribosome biogenesis, were identified in the *Arabidopsis* genome, whereas 27 and 29 were found in rice and maize, respectively (**Table 2**). A recent proteomic analysis in *Arabidopsis* identified 1,602 nucleolar proteins, and 519 potential RBFs (Palm et al., 2016). Among these RBFs, 31 were identified as DExD/H-box RHs (Palm et al., 2016). This research together with our database search indicated that over 20 DExD/H-box RHs might participate in ribosome biogenesis in plants, suggesting that many steps in their ribosome biogenesis are RHs dependent, as has been shown in human and yeast.

**Table 2:** List of rice, maize, and *Arabidopsis* DExD/H-RHs with homology to human and yeast DExD/H-box RHs which function as RBFs.

Human	Yeast	Rice	Maize	*Arabidopsis*
DDX3X	Dbp1p	OS03G0805200 C H	Zm00001d007757	AT3G58510 (AtRH11)^∗^
DDX3Y	Ded1p	OS11G0599500 D	Zm00001d007755	AT2G42520 (AtRH37)^∗^ C
		OS07G0202100	Zm00001d048924 C D H	AT3G58570 (AtRH52)^∗^ C
DDX5	Dbp2p	OS01G0197200 D	Zm00001d039452	AT1G55150 (AtRH20)^∗^
DDX17		OS01G0911100 C D	Zm00001d042416	AT5G63120 (AtRH30)^∗^ C
DDX10	Dbp4p	OS07G0517000	Zm00001d006497 D	AT5G54910 (AtRH32)^∗^ C H
DDX18	Has1p	OS03G0802700 H	Zm00001d013056 C	AT3G18600 (AtRH51)^∗^ C H
		OS06G0535100	Zm00001d023501	AT5G65900 (AtRH27)^∗^ H
DDX21	N/A	OS09G0520700 C D H	Zm00001d006160 C D	AT5G62190 (AtRH7) C
DDX50			Zm00001d021196	
DDX24	Mak5p	OS04G0510400 D H	Zm00001d003031 C	AT3G16840 (AtRH13)^∗^ C H
DDX27	Drs1p	OS12G0481100 H	Zm00001d006113 D	AT4G16630 (AtRH28)^∗^
			Zm00001d021127	
DDX31	Dbp7p	OS05G0110500 D	Zm00001d010225	AT2G40700 (AtRH17)^∗^ C H
DDX41	N/A	OS02G0150100	Zm00001d047502	AT5G51280 (AtRH35)^∗^
		OS06G0697200 D		AT4G33370 (AtRH43) C
DDX47	Rrp3p	OS03G0669000 (TOGR1) D H	Zm00001d014787 C	AT5G60990 (AtRH10)^∗^ H
		OS07G0660000		
DDX48	Fal1p	OS01G0639100	Zm00001d018542	AT3G19760 (AtRH2)^∗^
		OS03G0566800	Zm00001d051840	AT1G51380 (AtRH34)^∗^ C H
DDX49	Dbp8p	OS07G0633500	Zm00001d022246	AT1G16280 (AtRH36) C
DDX51	Dbp6p	OS02G0795900 H	Zm00001d018375	AT4G15850 (AtRH1)
DDX52	Rok1p	OS07G0647900 H	Zm00001d022356	AT3G09720 (AtRH57)^∗^ H
			Zm00001d022360 C D	
DDX54	Dbp10p	OS08G0416100 C H	Zm00001d050315	AT1G77030 (AtRH29)^∗^ C
DDX55	Sbp4p	OS01G0164500 D H	Zm00001d039746	AT5G05450 (AtRH18)^∗^
				AT1G71370 (AtRH49) S
DDX56	Dbp9p	OS03G0728800 H	Zm00001d013358	AT4G34910 (AtRH16)^∗^ C H
DHX15	Prp43p	OS03G0314100 D	Zm00001d028923	AT3G62310 (DEAH2)^∗^
			Zm00001d047601	AT2G47250 (DEAH3)^∗^
DHX37	Dhr1p	OS02G0736600	Zm00001d017967 D	AT1G33390 (DEAH13)^∗^ C
SkiV2L2	Mtr4p	OS12G0279000 D H	Zm00001d045590 D	AT1G59760 (AtMTR4)^∗^

*The amino acid sequences of human and yeast DExD/H-box RHs were BLAST-searched against the protein databases of *Arabidopsis*, rice, and maize available at *Ensembl Plants* (http://plants.ensembl.org). The protein sequences showing the highest similarity were considered as homologs. Data for abiotic stress-responsiveness were obtained from eFP Brower (http://bar.utoronto.ca/efp_arabidopsis/) for *Arabidopsis* and *GENEVESTIGATOR* (https://genevestigator.com) for rice and maize. The genes showing a fold change >2 are indicated as C, cold inducible; D, drought inducible; S, salt inducible; and H, heat inducible following the annotation numbers. Asterisk “^∗^” indicates the *Arabidopsis* nucleolar DEAD-box RHs reported as human and yeast RBFs orthologs (Palm et al., 2016)*.

Recent studies on AtRH7 and TOGR1 have suggested that stress inducible RBF-type RHs play an important role in modulating plant stress adaptation (Huang et al., 2016; Liu et al., 2016; Wang et al., 2016). This led us to search two plant expression databases, eFP Brower^2^ and GENEVESTIGATOR^3^, to analyze the stress-responsive expression pattern of previously identified candidates. As shown in **Table 2**, many RH genes are potentially up-regulated by at least one type of stress, implying that these DExD/H-box RHs may participate in plant stress responses. Ribosome biogenesis is known to be highly coupled with stress stimuli (Boulon et al., 2010); DExD/H-box RHs are critical players in this life process. Therefore, it will be interesting to investigate how these helicases modulate rRNA maturations and how they contribute to plant morphogenesis under stress conditions in future studies.

## Conclusion and Perspectives

This mini-review has focused on the DExD/H-box RHs which function in pre-rRNA processing in plants. Recent studies have revealed physiological roles of these RHs in plant reproduction, development, and stress responses. However, their precise molecular functions in rRNA biogenesis still remain unclear. Thus, in future studies, it will be necessary to investigate which steps of the pre-rRNA processing these DExD/H-box RHs are involved in, and how they recognize their target rRNA sequence. In human cells, single DExD/H-box RHs function in a variety of steps in ribosome biogenesis. For instance, DDX47 has a potential role in rRNA transcription in addition to pre-rRNA processing (Sekiguchi et al., 2006; Zhang et al., 2011); DDX21, a homolog of AtRH7, not only regulates pre-rRNA processing but also modulates transcription of rRNA, snoRNAs and RP mRNA (Henning et al., 2003; Calo et al., 2014; Xing et al., 2017). Therefore, it will be interesting to determine if plant DExD/H-box RHs participate in the transcription of ribosome biogenesis components. Moreover, many DExD/H-box RHs are still uncharacterized but are predicted to be involved in ribosome biogenesis (**Table 2**). Therefore, future work should be focused on characterizing these potential candidates and revealing their functions in plant growth and stress adaptation. Together, these investigations will provide further insights into the complexity of plant ribosome biogenesis and the intrinsic connection between ribosome biogenesis and plant physiological processes.

## Author Contributions

All authors listed have made a substantial, direct and intellectual contribution to the work, and approved it for publication.

## Conflict of Interest Statement

The authors declare that the research was conducted in the absence of any commercial or financial relationships that could be construed as a potential conflict of interest.

## Footnotes

1 http://plants.ensembl.org

2 http://bar.utoronto.ca

3 https://genevestigator.com
